# Follow Up Data of MRI-Visible Synthetic Meshes for Reinforcement in Large Hiatal Hernia in Comparison to None-Mesh Repair—A Prospective Cohort Study

**DOI:** 10.3389/fsurg.2019.00017

**Published:** 2019-04-16

**Authors:** Dirk Weyhe, Uwe Klinge, Verena Nicole Uslar, Navid Tabriz, Alexander Kluge

**Affiliations:** ^1^School of Medicine and Health Sciences, Pius-Hospital Oldenburg, University Hospital for Visceral Surgery, Medical Campus University of Oldenburg, Oldenburg, Germany; ^2^Clinic for General, Visceral and Transplant Surgery, University Hospital RWTH Aachen, Aachen, Germany; ^3^Institute of Diagnostic and Interventional Radiology, Pius-Hospital Oldenburg, Oldenburg, Germany

**Keywords:** dysphagia, hiatal hernia, MRI-visible mesh, DynaMesh, quality of life, early results, mid-term results

## Abstract

**Background:** Mesh augmentation for large hiatal hernia is still controversial because of high alleged risk of chronic reaction or shrinkage of mesh orifice surrounding the esophagus. The aim of this cohort study was to develop and establish an image analysis scheme, including 3D reconstruction, for MRI-visible meshes (DynaMesh®) to measure postoperative mesh shrinkage in order to observe potential complications.

**Methods:** Between 12/2012 and 10/2016, *n* = 33 patients underwent surgery to correct symptomatic hiatal hernia (implantation indicated: *n* = 18). Intraoperative measurement of the hiatal surface area (HSA) > 5 cm^2^ was indication for mesh implantation. Early postoperatively, and during long-term follow-up, MRI was performed and patients filled out the gastrointestinal quality of life index (GIQLI score).

**Results:** Follow-up rate was 76% (*n* = 25/33). Overall recurrence rate was 4% (1/25). No other patient showed reflux or dysphagia symptoms. Mesh related complications were not observed during follow-up period. Median GIQLI score of patients with mesh was 123 (range: 67–144), and 93 (52–141) for patients without mesh. Comparison of early and mid-term postoperative MRI for patients with mesh showed changes in mesh orifice size of 3% (corresponding to a slight increase in size of about 6 mm^2^) without any significant correlations with BMI, HSA, or patient age.

**Conclusion:** We established an image analysis and 3D reconstruction scheme for MRI visible meshes in hiatal hernia repair. MRI images of normal clinical quality are sufficient for this analysis. Mesh orifice size in MRI-visible meshes does not seem to change at a clinically relevant level in the small cohort observed here. Further studies of large cohorts are necessary to establish if HSA >5 cm^2^ could be a suitable measure for indication of mesh implantation.

## Introduction

Hiatal hernias can for instance result from advanced age and adiposity (1), possibly due to a combination of insufficient hiatal fixation of the cardiac region, decreasing elasticity of the phrenoesophageal membrane, and elevated intra-abdominal pressure. The extent to which the gastroesophageal junction is displaced is the basis for hiatal hernia classification (2–4). Sliding hernias (type I), paraesophageal hernias (type II), and mixed hernias (type III) have been described; type IV hernias are extreme defects in the diaphragm which involve herniation of other organs in addition to the stomach, such as the small intestine or colon, and in extreme cases the pancreas and spleen.

It is possible to diagnose hiatal hernia and/or GERD via MRI (5). However, patients with intrathoracically displaced abdominal organs can remain asymptomatic for many years before the typical hiatal hernia symptoms appear, such as reflux, dysphagia, regurgitation and post-prandial heart/circulation problems, or even cardiac arrhythmia and hemorrhagic anemia. The prevalence of complex or large hiatal hernias is unknown. Even when the literature describes small, medium, and large hiatal hernias, classification is impossible without a definition of the size of the hernial orifice. This lack of a clear definition of hernial size makes it nearly impossible to compare clinical results.

Symptomatic hernias must be treated surgically, but some surgical details remain controversial. One unresolved issue at the center of the debate is the use of prosthetic mesh or biological membranes to reinforce the esophageal hiatus. Compared to simple sutures, reinforcement with hiatoplasty has achieved significant reductions in recurrence rates in the medium and long term. Using sutures alone, recurrence rates of up to 42% have been described (6). Systematic reviews and meta analyses have established that in the short term recurrence rates well under 10% can be achieved through mesh reinforcement (7). It should be noted, however, that the discussion about recurrence rates is complicated by the fact that there are different definitions of recurrence, ranging from radiologically proven recurrences that are typically small and not symptomatic to clinically relevant symptomatic relapses. It is unclear, however, whether a circular or a partial augmentation of the esophageal hiatus is more appropriate. Hiatoplasty may carry the risk of a chronic reaction to the uncoated polypropylene or polyester polymers, which is associated with a risk of hollow organ erosion and intestinal fistulas and has been described primarily in small case series (8–10). A further potential complication is shrinkage of the mesh orifice surrounding the esophagus followed by esophagogastric stenosis and dysphagic complaints (11). New innovations in MRI-visible mesh enable long-term observation of the healing process and potential shrinkage of the implant, and these observations can be correlated with the clinical results (12).

### Aim of the Study

A technique is used for measuring hiatal hernia size intraoperatively, which, to our knowledge, is not implemented in other clinical studies so far (13). Therefore, the first aim of this study was to assess whether this technique provides a workable measure to standardize indication of mesh implantation. Secondly, a new MRI image analysis scheme was developed, and the feasibility of the new scheme was tested in this prospective cohort study. Because of the ongoing debate regarding the safety of mesh implantation, establishing an analysis scheme to assess the suitability of mesh implants in hiatal hernia repair by using a MRI-visible mesh, and thus being able to monitor the healing process, seems of utmost importance.

## Materials and Methods

### Study Design

Between 12/2012 and 10/2016, all patients undergoing surgery to correct a symptomatic hiatal hernia in the University Hospital for Visceral Surgery (Medical Campus University of Oldenburg, Pius-Hospital Oldenburg) were included in this prospective observational study (*n* = 33). Volume reflux and radiologic confirmation of the hiatal hernia were main criteria for surgery indication. After informed consent, patients were recorded in an in house registry and examined systematically, all in accordance with the Declaration of Helsinki, and with an ethical votum approving the experimental protocol obtained during ongoing trial (12/2015; Medical Ethics Committee of the University of Oldenburg No: 016/2015). In all cases, a CT or MRT was performed pre-operatively as part of the outpatient or stationary diagnosis used for surgical planning. Following the recommendations of Granderath and Pointner intra-operatively, a hiatal hernia surface area (HSA) > 5 cm^2^ was considered a medical indication for mesh reinforcement (14). In *n* = 18 patients, implantation of an MRI-visible mesh (DynaMesh®) was performed. In *n* = 15 patients, no mesh was implanted because either the HSA was smaller than 5 cm^2^ or the patient explicitly refused a mesh as part of the patient education. Within 1 week post-surgery (early post-operative) and again after a median follow-up of 1 year (range: 10–27 months; mid-term post-operative), in patients with mesh implant the position of the mesh was assessed using MRI. The size of the mesh orifice surrounding the esophagus was determined using a newly developed image analysis scheme. In addition, all patients filled out a written questionnaire [gastrointestinal quality of life index (GIQLI) score; appendix], which assesses quality of life.

### DynaMesh

The synthetic mesh is composed of polyvinylidenfluoride (PVDF). Iron particles incorporated into the mesh enable visualization by MRI. The mesh and the central opening are squarely constructed to ensure high stability under strain (Supplementary Figure 1).

### Surgical Technique

**Figure 1:**
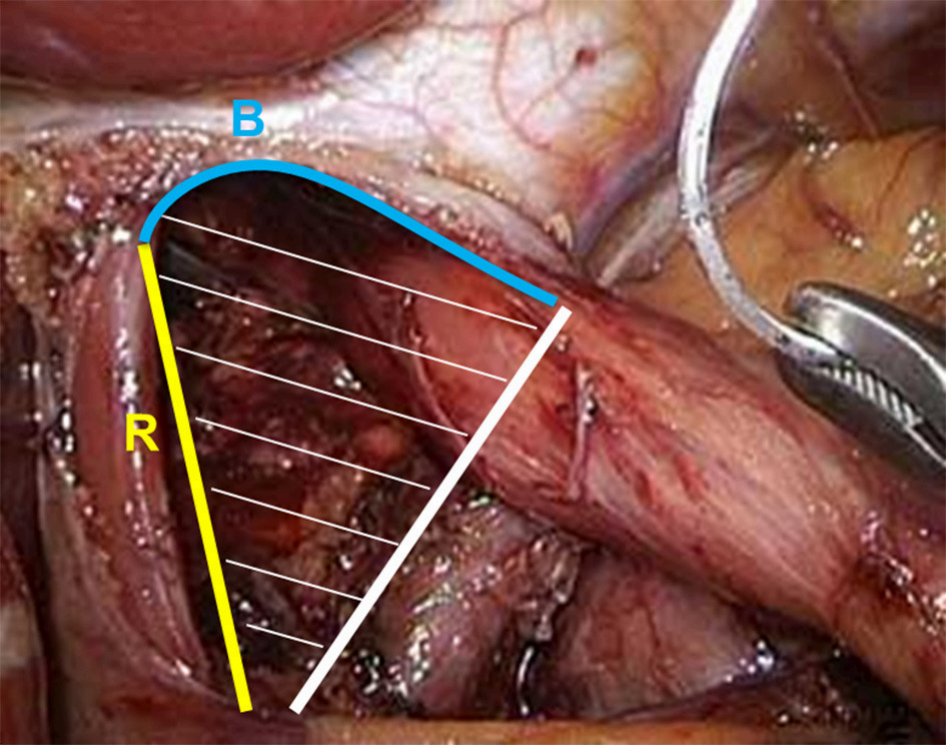
Measurements of the HSA according to Granderath (14): the length of the crural commissure (R, yellow) and the length of the diaphragmatic commissure (B, blue; following the curve of the commissure) are measured and the HSA can then be calculated as B * R/2 or estimated by plotting B against R (see also Supplement Figure 2).

Patients were positioned in the beach chair position. The abdominal gas insufflation pressure was standardized at 12 mmHg. Retraction of the left hepatic lobe and exposure of the hernial orifice; repositioning of herniated tissue to the abdomen where possible. Dissection of the pars flaccida and preparation of the diaphragm crura in a clock-wise fashion, followed by preparation and separation of the intrathoracic hernial sack. The esophagus is mobilized far within the thorax (minimum: 6 cm). To estimate the size of the hernia, the length of the crural commissure (R) and the length of the diaphragmatic commissure (B; not measured as a straight line between both crural edges, but rather following the curve of the commissure; see also Figure 1) are measured, and the HSA was then either calculated as B^*^R/2 or plotted (Supplementary Figure 2) (13).

The dorsal hiatoplasty was performed using three intracorporeal polyester threads knotted using single sutures. Depending on the anatomy and size of the hernia, an additional ventral suture may have been applied. For HSA >5 cm^2^ implantation of a 7 × 12 cm MRI-visible PVDF polymer (DynaMesh® MRI-visible) was performed. The slit mesh was pulled under the esophagus from left to right and placed such that the slit was positioned in the upper left quadrant.

The central mesh opening was placed such that it neither touched nor constricted the esophagus. The fixation was carried out near the muscular diaphragm crura with absorbable tackers (Absorbatack, Covidien®). Where necessary, an absorbable suture in the area of the central tendon was supplemented. Additional fixation was not necessary, since the intraabdominal pressure provided *in situ* fixation, holding the mesh in place. In all cases, we performed a 360° fundoplication. This was to ensure a uniform distribution of pressure on the reconstructed hiatal esophagus. To this end, the gastric fundus was mobilized at least 12–14 cm in order to ensure a tension-free wrap. The fundus wraps were stabilized using three polyester sutures, whereby the middle suture also included the cardia region to prevent pouch slipping.

### MRI Follow-Up

Early and mid-term post-operatively, the position of the mesh was controlled using MRI. All MRI examinations were performed using a 3T MRI Siemens Magnetom Verio (Siemens Healthcare, Erlangen, Germany). Patients were positioned in the supine position. MRI sequences of the cardiac region in the parasagittal plane were oriented parallel to the axis of the stomach and perpendicular to that plane, using the following parameters: T1-weighted 2d spoiled gradient echo sequence, TR 84 ms, TE 2.46 ms, FOV 380 mm, resolution 240 ^*^ 320, slice thickness 4 mm, 25 slices, acquisition time 18 s within a single breath hold. One additional sequence was performed allowing free breathing using a navigator gating technique to detect diaphragmatic motion. The following sequence parameters were used: T2-weighted 2d spoiled gradient echo sequence, TR 4,000 ms, TE 2.46 ms, FOV 380 mm, resolution 240 ^*^ 320, slice thickness 5.5 mm, 18 slices, acquisition time 3.5 min. Total examination time was <10 min.

### Image Analysis

MRI images were inspected visually to detect the implanted net. Due to the susceptibility induced signal voids the visible mesh produces signal loss expressed by a dark rim at its position on all sequences. The position of the cardia was assessed visually as well to assess for hernia relapse. To quantify the change in size of the esophageal orifice mesh opening, the mesh orifice size at 1 week and at 1 year post-surgery was compared. A combined graphical-numerical measurement procedure was applied.

#### Numerical Procedure

**Figure 2:**
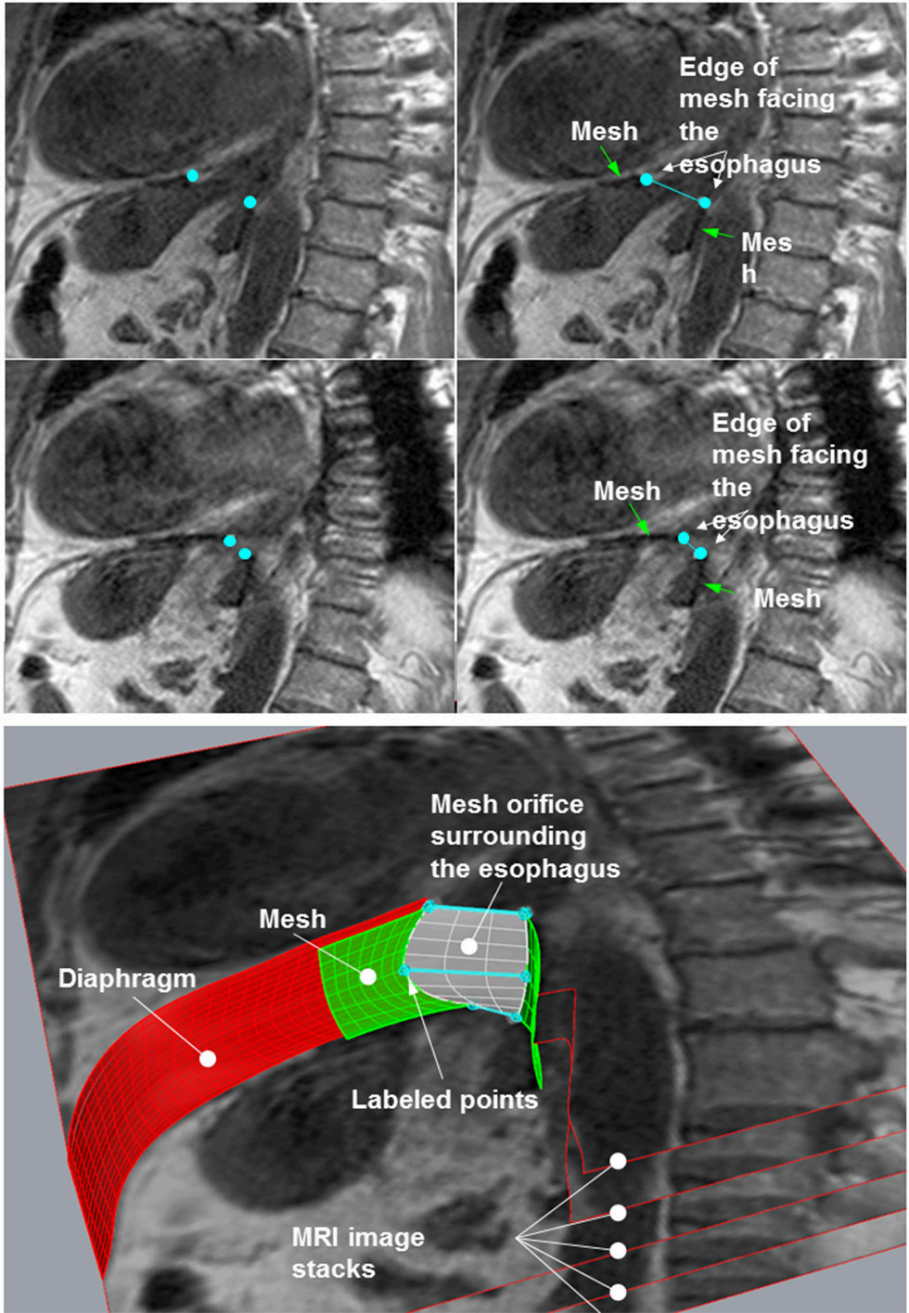
**(Upper)** Two images from one sequence used for analysis. Left column: the mesh edge closest to the esophagus was labeled using ImageJ (small, light gray square dots). These images were saved for control purposes. Right column: the same images with descriptions. **(Lower)** Calculation of the mesh orifice by stacking the images and generating a skewed plane between the marked points.

The MRI sequences were examined and the one in which the mesh was best captured was selected. This sequence was then loaded using the image analysis program ImageJ (15). In the images, the points on the edge of the mesh closest to the esophagus were labeled and their coordinates were stored in a table: these points lay on the edge of the hernial opening (Figure 2, upper panel). The images with the labeled points were saved and served as controls. The tables with the coordinates were used for numerical evaluation of the opening using an Excel macro in Visual Basic for Applications (VAB).

#### Reconstruction of the Opening by Image Stacking

For reconstruction of the hernial opening, the MRI images were arranged in a stack (Figure 2, lower panel). The distance between the images was set to the spacing between slices of the MRI sequence. Corresponding labeled points of adjacent images were connected by straight lines resulting in two polygons, which represent the edges of the hernial opening. The polygons were filled out by hyperbolic paraboloid surfaces between corresponding lines of the two polygons (white plane in Figure 2, lower panel).

Since the area of the constructed surfaces cannot be determined using closed integration, numerical integration was employed. The Excel macro developed for this purpose was validated previously (15). Using a CAD program, the hyperbolic paraboloid surfaces were graphically generated as a reference. The surface areas calculated by the CAD program were very precise and had a margin of error of at most ±10^−6^ mm^2^. As the number of data points in the numerical integration increased, the Excel macro values converged with the reference values. The calculated data may then be used to create a 3D schematic representation of the mesh *in situ* (see Video in Supplementary Material).

### Statistical Methods

Because of the pilot study character of this study, no sample size calculations were implemented and only descriptive statistics were used, with the following exceptions. Spearman rank correlations were calculated for early and mid-term post-operative esophageal orifice size, and the change in size vs. age, BMI, and initial HSA value, respectively. All data was analyzed descriptively using IBM SPSS version 23 (16).

## Results

**Figure 3:**
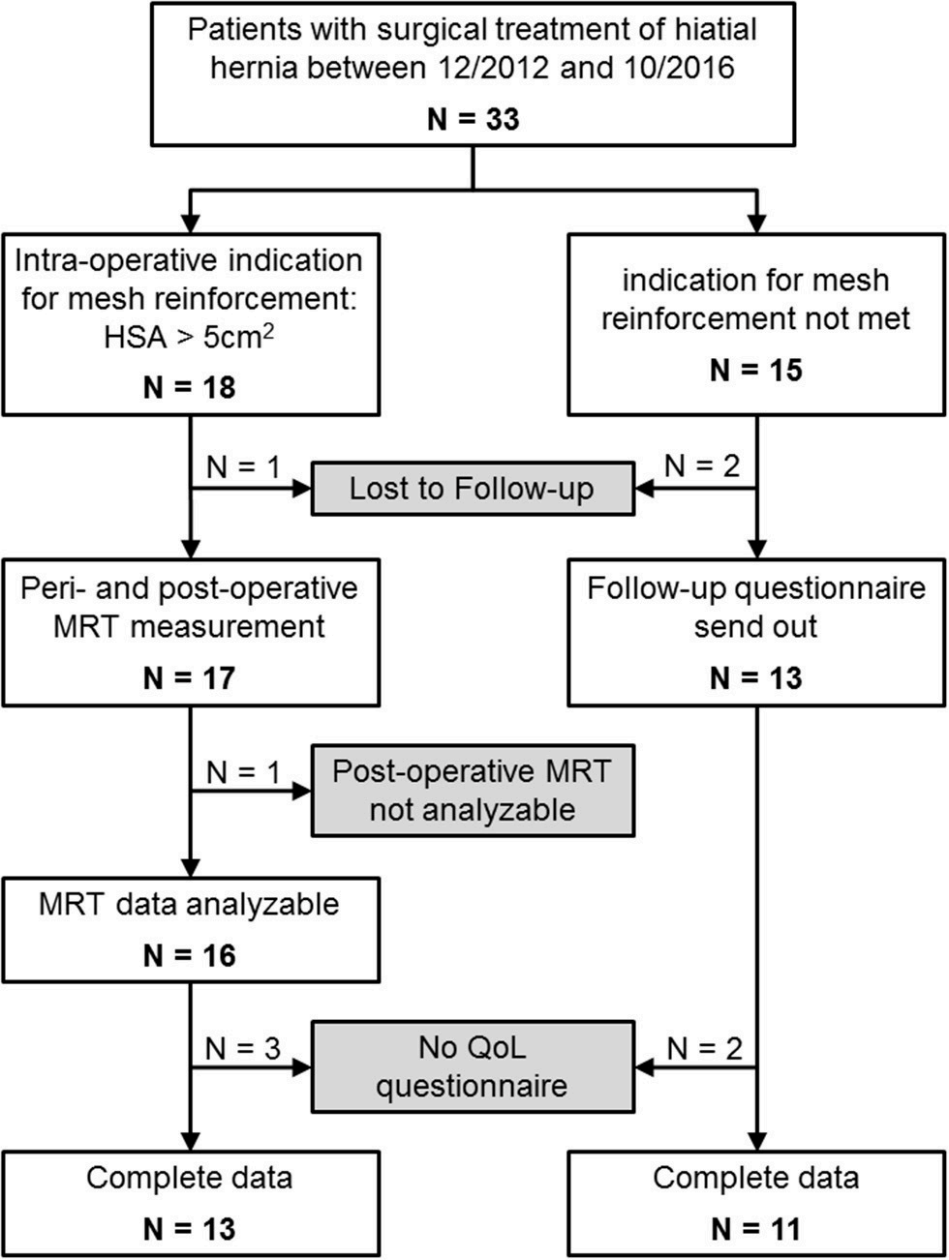
Flow chart of the study design.

From January 2013 through December 2014, *n* = 33 were included in this study because of surgical treatment of a hiatal hernia (see Figure 3 and Table 1). In *n* = 18 patients the indication for mesh reinforcement (HSA > 5 cm^2^) was met intraoperatively. Perioperative MRI was done in all patients with mesh implant. MRI follow-up after an average of 1 year succeeded in 89% of those cases (16/18). Out of the 16 patients with MRI follow-up three were not willing to fill out the GIQLI questionnaire, because of the rather personal character of the questionnaire. Thus, complete follow-up data including GIQLI score was obtained in 72% of all cases (13/18).

**Table 1:** Patient charactersitics of all patients with and without mesh augmentation.

**Pat. no**	**ASA**	**Hiatal hernia type**	**HSA**	**Esophageal orifice peri-op [mm^**2**^]**	**Esophageal orifice post-op [mm^**2**^]**	**Change in esophageal opening size [%]**	Post-op disorders^*^	**GIQLI score**
1	3	1	5.0	264	222	−16	No	82
2	3	3	5.5	245	182	−26	No	98
3	2	4	7.4	228	241	6	Recurrence	Not measured
4	3	4	7.5	388	407	5	No	134
5	3	3	7.6	123	133	8	Sporadic	67
6	3	3	8.2	206	222	8	Sporadic	88
7	2	2	8.2	229	227	−1	No	134
8	3	4	8.9	95	No second MRI		Lost to follow-up	Not measured
9	3	2	9.1	415	361	−13	No	133
10	2	3	9.3	438	474	8	No	Not measured
11	2	3	9.8	141	160	13	Sporadic	95
12	3	4	10.3	262	246	−6	Sporadic	82
13	2	3	10.6	297	261	−12	No	123
14	2	3	10.7	212	273	29	Frequent	Not measured
15	3	1	11.2	129	Not analyzable		No	Not measured
16	2	3	13.2	124	132	6	No	126
17	1	3	17.5	350	352	1	No	133
18	2	3	31.6	291	295	1	No	140
19	1	1	1.5				Sporadic	78
20	2	1	2.3				No	Not measured
21	3	1	2.3				Frequent	52
22	3	1	2.6				No	95
23	2	2	2.7				Frequent	114
24	3	3	3.1				Sporadic	78
25	2	1	3.3				Lost to follow-up	Not measured
26	3	1	3.4				Frequent	64
27	2	1	3.9				Sporadic	90
28	3	1	4.0				Lost to follow-up	Not measured
29	2	1	4.5				Sporadic	120
30	3	1	5.0	No mesh despite indication			No	141
31	2	1	5.4	No mesh despite indication			No	112
32	2	1	6.3	No mesh despite indication			Frequent	Not measured
33	2	1	7.6	No mesh despite indication			Frequent	108

* *Post-operative disorders include instances of pain, regurgitation, reflux, or recurrence*.

For *n* = 15 no mesh augmentation was performed, either because the induction of an intra-operative HSA > 5 cm^2^ was not met (*n* = 11; patients no. 19–29 in Table 1), or patients explicitly rejected mesh augmentation (*n* = 4). All data was gathered for these four patients. However, they will be discussed separately. In the non-mesh group, *n* = 2 patients were lost to follow-up. The remaining 13 patients received a questionnaire via mail 1–3 years postoperatively. All those patients gave at least a short comment as to their general health and possible disorders related to the surgery. *N* = 11 patients sent back the completed questionnaire.

### Preoperative Clinical Results

The overall gender ratio of the *n* = 33 patients was 3:2 (female:male). In the group with mesh the gender ratio was 5:1 (15:3 female:male), and in the group without mesh almost 1:1 (8:7). The age difference between the mesh and the non-mesh group was significant [mesh group: median 69.5 years, range: 51–86 years; non-mesh group: 55 years; range: 26–76 years; *t*_(31)_ = 3.122; *p* = 0.004]. The median BMI was 28.9 (range: 18.0–37.1). There was no difference regarding BMI between both groups. Twelve percent of all patients (4/33) reported nicotine use. Pre-operatively, about 80% of all patients complained of reflux, often in combination with regurgitation (about 60%) and dysphagia (see also Supplementary Figure 3). In general, a higher percentage of patients who later received a mesh suffered from any of the symptoms they were enquired about. However, this is not significant. Most patients in the non-mesh group presented with a type I hiatal hernia, whereas in the mesh group 56% of the cases the hernia was of type III (Supplementary Figure 4).

### Results of the Perioperative and Long-Term MRI Examinations

An early post-operative control (median: 6 days, range: 2–63 days) was carried out in 100% of the patients receiving mesh augmentation (18/18). In all cases, correct implant position with a completely subphrenic cardia was observed. The mid-term post-operative control was carried out in 94% of the patients (17/18; *n* = 1 patient lost to follow-up). Visual inspection allowed the detection of the mesh in 100% of examinations. However, in *n* = 1 patient mid-term post-operative MRI picture quality was not sufficient to allow for further image analysis. Thus, the precise position of the central opening could be determined visually in 94% of all patients with mesh implant (18/18 early post-operatively; 16/18 at the later post-operative measurement). Further analysis only regards all 16 patients for whom both MRI measurements produced analyzable data. 3D reconstruction was possible in all those cases (see also Supplementary Video).

**Figure 4:**
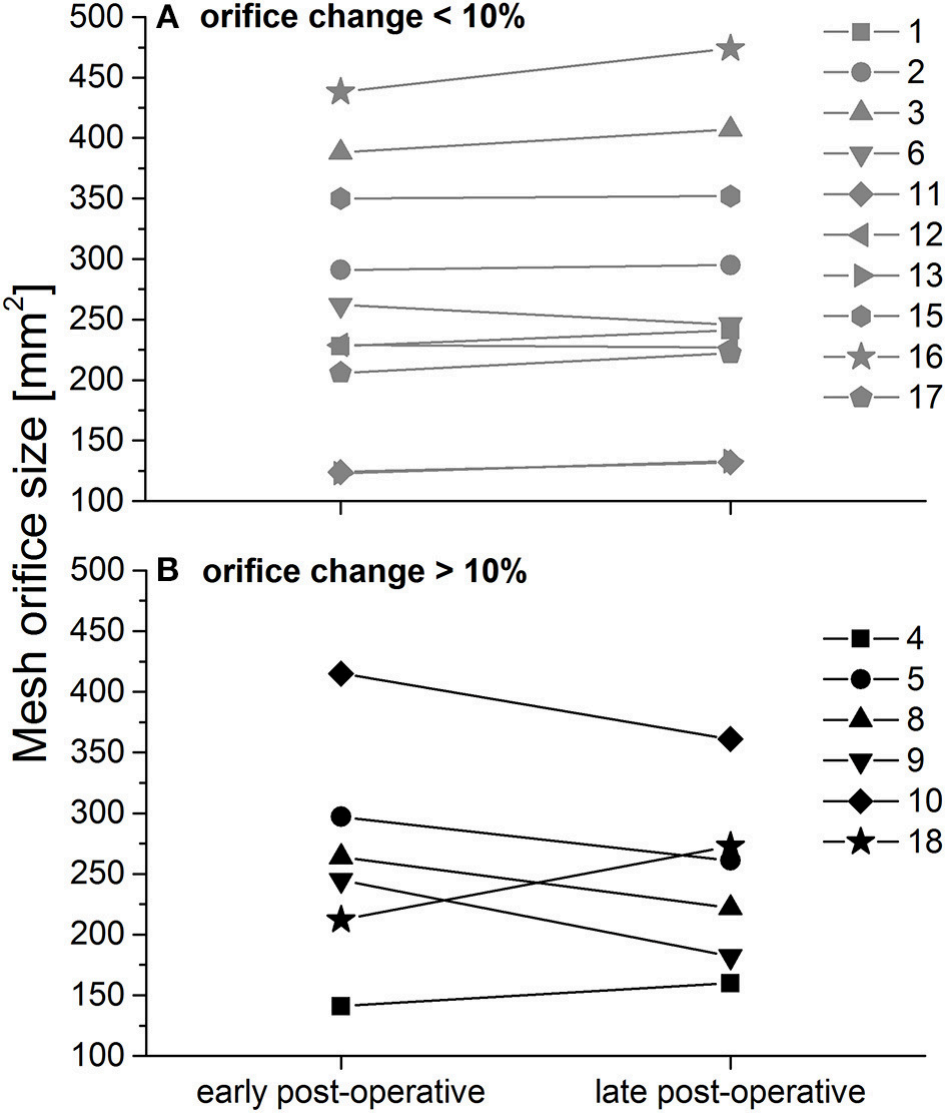
Calculated peri- and post-operative esophageal orifice size. **(A)** Patients with size change < 10%; **(B)** Patients with size change > 10%, as published in (4) with permission.

Median change in the size of the esophageal mesh orifice between early and mid-term post-operative MRI measurement was 3% (corresponding to a slight increase in size of about 6 mm^2^) with a range between 26% decrease and 29% increase (Figure 4). This corresponds to a maximum change in mm^2^ between +63 mm^2^ (decrease) and −61 mm^2^ (increase). Overall, the changes may be regarded as minimal, which is supported by the significant correlation between early and mid-term post-operative orifice size (Spearman rank; *r* = 871, *p* << 0.0001). There were no other significant correlations of the early or mid-term post-operative mesh orifice size, or of the size change with BMI, initial HSA, or patient age (Spearman rank correlation; *p* > 0.262 in all comparisons).

In 63% of all patients (*n* = 10) changes were below 10% (corresponding to changes of up to 36 mm^2^; Figure 4, upper panel). In 37% orifice size changed by more than 10% (corresponding to changes of up to 63 mm^2^; Figure 4, lower panel). Note that mid-term post-operative mesh orifice sizes in patients 4 and 18, who showed the smallest early post-operative orifice size, increased, while mid-term post-operative orifice size decreased in patients with larger initial orifice size. In those patients with large changes, neither elongation nor shrinkage was associated in any way with BMI or intra-operative HSA.

### Postoperative Clinical Results

**Figure 5:**
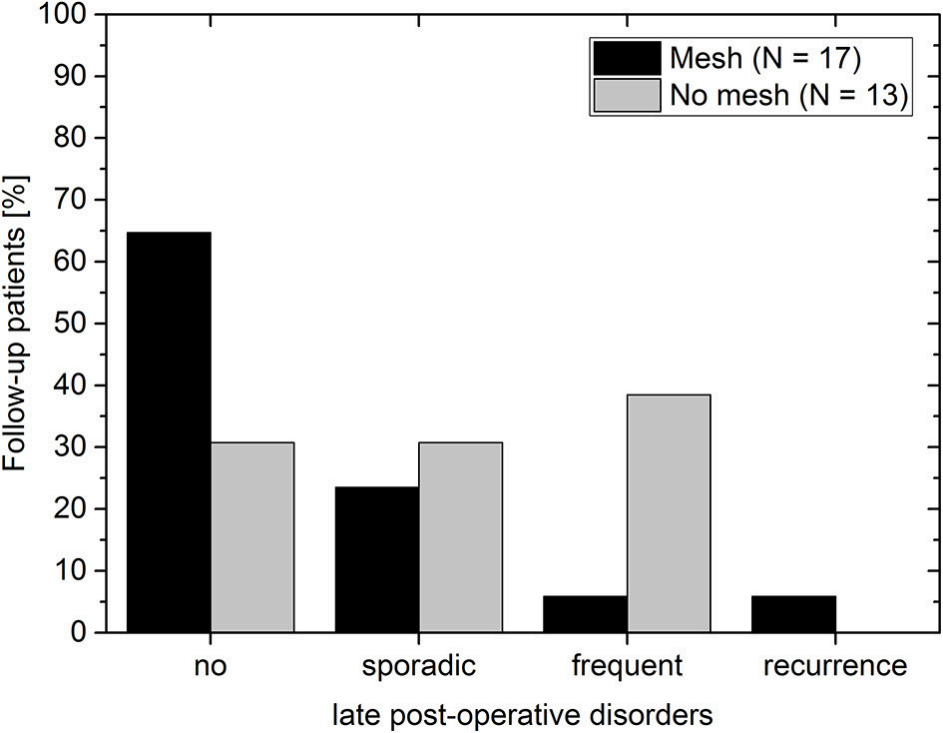
Post-operative disorders at the late follow-up for patients with mesh (black bars) and without mesh (gray bars). Post-operative disorders include instances of pain, regurgitation, or reflux.

The overall recurrence rate was 4% (1/25). None of the 17 follow-up patients of the mesh group complained post-operatively of dysphagia. Thus, the recurrence rate in the mesh group was 6% (1/17). It is assumed that for this patient the mesh size (7 × 12 cm) was too small for the hernial opening. Since the patient was clinically symptom-free, no further measures were undertaken. In the non-mesh group no symptomatic recurrences were observed. However, note that no imaging data was available at the mid-term follow-up to rule out any asymptomatic recurrences. In the mesh group about 30% (5/17) of the patients complained about sporadic (4/17) or frequent (1/17) incidences of pain, regurgitation or reflux (Figure 5). In the non-mesh group about 70% (9/13) of the patients complained about sporadic (4/13) or frequent (5/13) incidences of pain, regurgitation or reflux.

**Figure 6:**
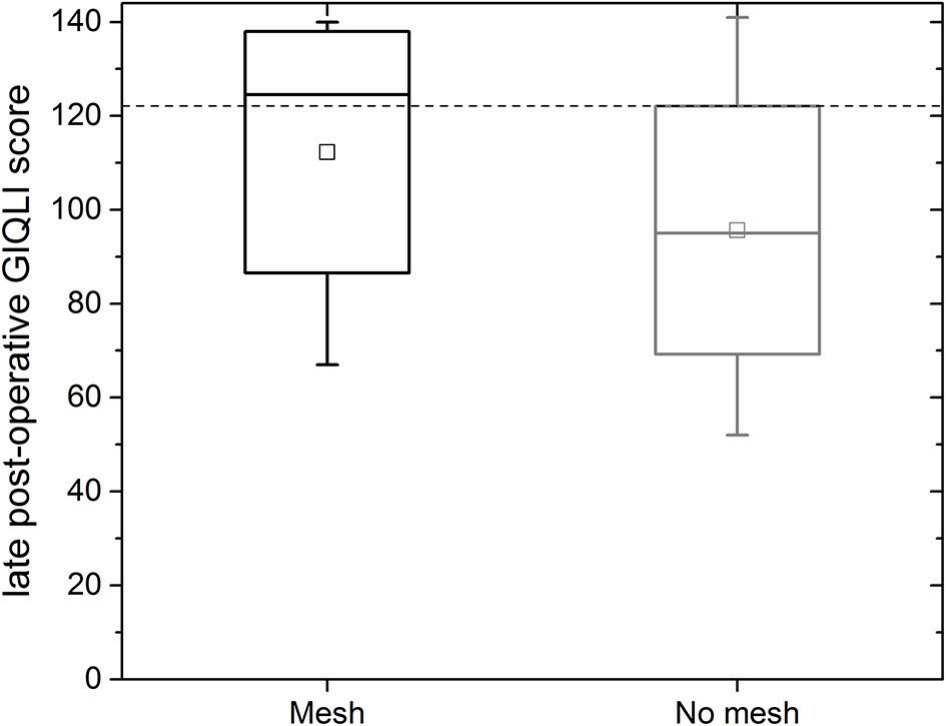
GIQLI score for all follow-up patients with mesh (black box, left) and for patients without mesh (gray, right); the GIQLI is scored by totaling the points from the survey. This figure shows the data for all patients as a median, quartiles and range; the dotted line indicates the average score of a healthy subject (123).

For the mesh group, the median gastrointestinal quality of life index (GIQLI) score was 123 out of 144 points (range: 67–140 points; see Figure 6); this corresponds to the score of a healthy person. The results of the GIQLI score did not depend on BMI, HSA, age, or the change in the size of the esophageal orifice between the early and mid-term post-operative MRI measurement. The same holds true for the non-mesh group. However, the median GIQLI score in the non-mesh group is a little lower (median: 95, range: 52–141). This difference between groups is not statistically significant.

## Discussion

Mesh reinforcement for prevention of hernia relapse in abdominal wall and inguinal hernia surgery is an integral part of the international guidelines with a very high level of evidence in both conventional and laparoendoscopic surgical procedures (17–19). The SAGES guidelines also state a considerably lower recurrence rate after mesh reinforcement of hiatal hernias. However, mesh implantation at the hiatal esophagus is not yet recommended by the guidelines, because of the high risk of complications and a lack of long-term data (20). Coated polymers appear to reduce these risks in experimental settings (21). PTFE, or combinations of PTFE and polypropylene, however, do not yield a complete integration of the mesh and are associated with strong mesh shrinkage (22–24). When biological membranes are used, a high recurrence rate and fibrosis-induced dysphagia are observed. Therefore, the IEHS Guideline explicitly recommends against using biological membranes for incisional hernia (25). All in all, the material-related complication potential seems exaggerated: when one examines the described complications in relation to application frequency, the rate of complication is only 0.8% (26). Also, according to the same analysis, polypropylene meshes and the completely absorbable polyglactin nets (Vicryl®) appear to have the lowest complication rates.

The structural stability of plastic nets has not been thoroughly investigated. In particular with respect to mesh shrinkage in the region of the hiatal esophagus, implantation stability is of particular relevance, since shrinkage can lead to post-operative dysphagia and hollow organ herniation. Shrinkage can be largely counter-acted through the structural stability of the implant. Studies using animals showed that the elongation and deformation of plastic nets larger than 50 Nm are a predictor for shrinkage *in vivo* (27).

In the present study, both the clinical and the radiological results show that no relevant decrease in mesh orifice size occurred during follow-up time, which would have been an indicator for potential future dysphagia. On the contrary, in the majority of our patients, an increase in mesh orifice size was observed. It should be noted, however, that the differences between the first, early post-operative measurement and the second, later post-operative measurement, was so small that it may be due to measurement uncertainty of the newly developed MRI image analysis scheme. There were no indications of arrosions or severe complications. The one early post-operative relapse observed could be attributed to an inadequate mesh overlap for a patient with hernia type IV (partial dislocation of the stomach) and a large HSA. In the future, the manufacturer should offer larger implants for these cases. According to our observations, the crural fixation of the mesh is completely adequate for holding the mesh *in situ* during the integration phase.

A limitation of the study can certainly be seen in the small number of cases. Since the MRT visible meshs have not been on the market for very long and large numbers of cases are therefore not possible, this study can at least provide some initial indications of the usefulness of this technology. However, multi-center or registry studies still have to be carried out in order to generate large numbers of cases. Aside from this, we did not obtain a pre-operative GIQLI score. Thus, a pre/post comparison is missing in this instance. However, long-term quality of life is markedly higher in the mesh group as compared to the non-mesh group, and is better or comparable to literature [e.g., (28–30)]. An additional limitation of this study are missing image-based diagnostics for the follow-up of the non-mesh group, for whom the follow-up entailed only clinical diagnostics and no barium swallow and/or MRI as compared to the mesh group. In the non-mesh group we did not observe any symptomatic recurrence. This might be due to the fact that a HSA > 5 cm^2^ is a suitable cut-of size for mesh indication. Or, since it is known that recurrence rates differ depending on the type of diagnostics done during follow-up (31) small, asymptomatic recurrences were simply not detected in the non-mesh group. It should be noted that, even with this uncertainty accounted for, our recurrence rate is at least comparable to those found in different meta-analyses (26, 31–33). Connected to this is also the fact that we have defined a hernia as large -and thus a mesh augmentation as indicated- if the HSA was 5 cm^2^ or larger. This cut-off is based on our own experience and literature proposals (13, 14) and has not yet been systematically investigated. Again, this can only be done within the framework of larger studies.

Analysis of mid-term post-operative incidences of pain, regurgitation, reflux, and recurrence further strengthen our hypothesis that the limit of HSA > 5 cm^2^ appears to be a suitable cut-off for mesh implantation since almost 90% of all patients in the mesh group report no or only sporadic problems. By contrast, two out of the four patients receiving no mesh implant for various reasons despite meeting the indication criterium report frequent problems. Moreover, it is unclear why in general the non-mesh group reports more incidences of post-operative disorders than the mesh group. Therefore, a more detailed (multi-center) study analyzing outcome for various cut-off indication values is needed to further verify this data. A potential tool for conducting this research could be the EuraHS registry.

The novelty of our study thus lies in establishing a reliable analysis scheme for MRI-visible meshes translating 2D MRI data into 3D images of the mesh, and in providing first data over a longer follow-up time for MRI-visible meshes in hiatal hernia. For a reliable image analysis of the MRI images, these images must have the normal clinical quality, and there are no further or special requirements.

## Conclusion

The potential for relapse increases with hernia size. The intra-operative HSA measurement and entry into a coordinate system enables an intra-operative assessment of the need for mesh implantation. The limit of HSA > 5 cm^2^ is based on published recommendations (14). However, the exact cut-off for mesh indication remains unclear, especially since there are also patient-related factors to consider. Thus, a larger RCT and/or registry study will be needed to establish evidence-based recommendations for mesh implantation in hiatal hernia. Implantation of structurally stable meshes prevents clinically relevant shrinkage with accompanying dysphagia, and the relapse rate in the first year falls to <4% in this small cohort. The newly developed image analysis scheme for MRI-visible implants enables long-term visual control and 3D reconstruction of the mesh, that is minimally burdensome to the patient. To validate this approach, an international, multi-center, prospective register study will be carried out on the platform of the EuraHS-register (www.eurahs.eu).

## Previous Communication

D. Weyhe, V. Uslar, M. Hoffmann, M. Grewe, A. Kluge, Mesh in hiatal hernia repair without any clinical relevance of shrinkage or elongation—a prospective study (Abstracts Viszeralmedizin 2017; Best Poster Award). Innovative Surgical Sciences, 2017. 2(s1).

## Ethics Statement

Ethical votum approving the experimental protocol obtained during ongoing trial (12/2015; Medical Ethics Committee of the University of Oldenburg No: 016/2015).

## Author Contributions

Substantial contributions to the conception/design of the study were made by DW, UK, and AK. DW, VU, and NT were in charge of the acquisition of data. Analysis and interpretation of data were conducted by DW and VU. VU, DW, and NT wrote the main manuscript text. VU prepared all figures. All authors reviewed the manuscript.

### Conflict of Interest Statement

UK reports personal fees from expert testimony, and personal fees from consultant FEG, and grants from E-Mesh BMBF outside the submitted work; In addition, UK has a patent PVDF mesh with royalties paid. The remaining authors declare that the research was conducted in the absence of any commercial or financial relationships that could be construed as a potential conflict of interest.

## Supplementary Material

Supplementary Figure 1 Image of mesh. The iron particles cause the dark strands visible in the close-up.

Supplementary Figure 2 Determining the HSA according to Granderath (14): the length of the crural commissure (R) is plotted on the x-axis; the length of the diaphragmatic commissure (B; measured following the curve of the commissure; see also Figure 1) is plotted on the y-axis. The blue line depicts the values of B as a function of R for which HAS = 5 cm^2^ calculated as B ^*^ R/2. Data points above the blue line indicate an HSA > 5 cm^2^ and thus an indication for mesh reinforcement.

Supplementary Figure 3 Pre-operative symptoms of the patients that received a mesh implant (black bars; *n* = 18), and of patients that did not receive a mesh (gray bars; *n* = 15); multiple responses possible.

Supplementary Figure 4 Hernia type distribution according to Kahrilas (3). **(a)** Patients without mesh implant. **(b)** Patients with mesh implant.

Supplementary Video 1 3D reconstruction of patient specific mesh (Patient a).

Supplementary Video 2 3D reconstruction of patient specific mesh (Patient b).
