# Supplementary figures and images for: Follow Up Data of MRI-Visible Synthetic Meshes for Reinforcement in Large Hiatal Hernia in Comparison to None-Mesh Repair—A Prospective Cohort Study
Source: Front Surg. 2019 Apr 16;6:17. doi: 10.3389/fsurg.2019.00017 (PMC6477929; doi:10.3389/fsurg.2019.00017)

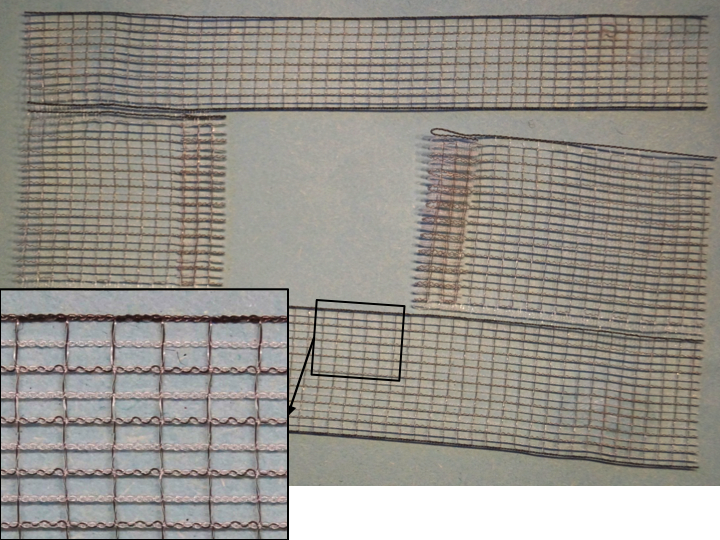

Supplement: Supplementary Figure 1 — Image of mesh. The iron particles cause the dark strands visible in the close-up. [file Image_1.TIF]

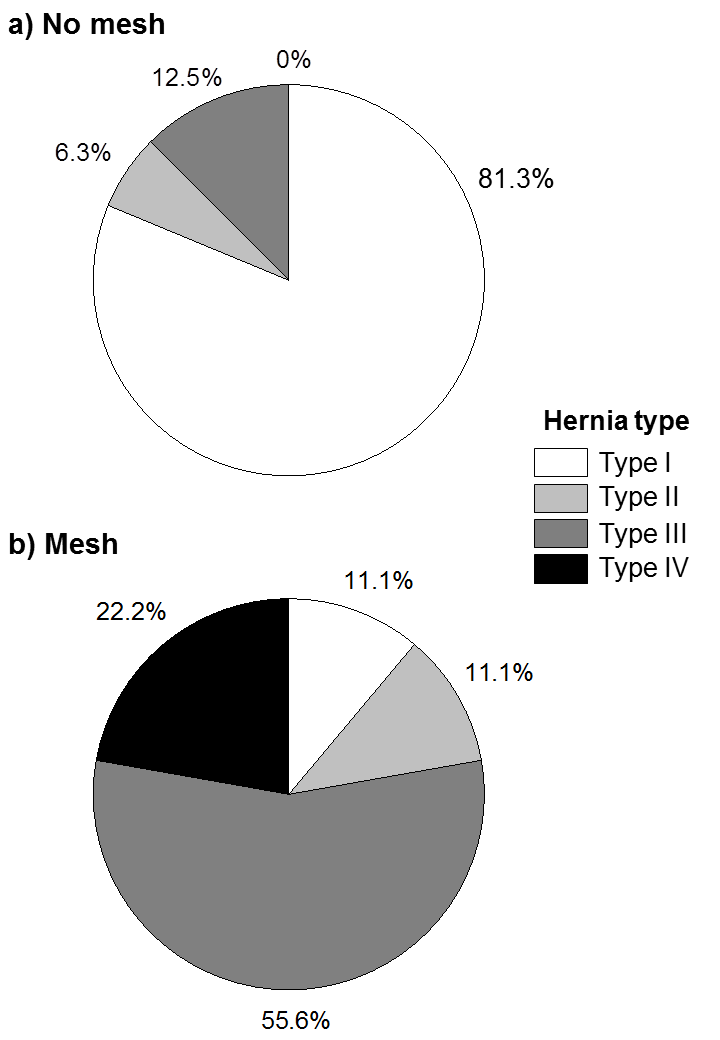

Supplement: Supplementary Figure 4 — Hernia type distribution according to Kahrilas (3). (a) Patients without mesh implant. (b) Patients with mesh implant. [file Image_4.TIF]
